# Endothelial Changes at Different Stages After Ischemic Stroke Contribute to the Regulation of Immune Cell Infiltration

**DOI:** 10.1111/cns.70456

**Published:** 2025-05-27

**Authors:** Xuejiao Dai, Xiaotao Zhang, Jiarui Chen, Qia Zhang, Huaming Li, An Ping, Yuchen Liu, Jingyi Zhou, Jianmin Zhang, Ligen Shi, Jianan Lu

**Affiliations:** ^1^ Department of Neurosurgery, Second Affiliated Hospital, School of Medicine Zhejiang University Zhejiang Hangzhou China; ^2^ Department of Health Management Center, Second Affiliated Hospital, School of Medicine Zhejiang University Zhejiang Hangzhou China; ^3^ Clinical Research Center for Neurological Diseases of Zhejiang Province Hangzhou China; ^4^ Brain Research Institute Zhejiang University Zhejiang Hangzhou China; ^5^ Collaborative Innovation Center for Brain Science Zhejiang University Zhejiang Hangzhou China; ^6^ State Key Laboratory of Transvascular Implantation Devices Hangzhou China

**Keywords:** endothelial cells, ischemic stroke, single‐cell RNA sequencing

## Abstract

**Aims:**

Following ischemic stroke, peripheral immune cell infiltration is characterized by myeloid cell predominance in the acute phase and lymphoid cell infiltration in the subacute to chronic phases. Endothelial cells, as a critical interface between the peripheral circulation and the brain, upregulate adhesion molecules to facilitate immune cell infiltration. However, it remains unclear whether endothelial cells exhibit functional differences at different stages after ischemic stroke and how these differences affect immune cell infiltration.

**Methods:**

We performed single‐cell RNA sequencing on peripheral immune and endothelial cells from Sham and middle cerebral artery occlusion (MCAO) mice at 3 and 14 days post‐MCAO. Subsequent analysis of the sequencing data, combined with flow cytometry and immunofluorescence staining, was used to investigate the relationship between endothelial cell changes at different stages of stroke and immune cell infiltration.

**Results:**

We observed that the infiltration capacity of peripheral immune cells did not significantly increase at different stages after MCAO. However, endothelial cells underwent significant changes. By Day 3 post‐MCAO, there was an increased proportion of venous endothelial cells with enhanced angiogenesis and adhesion functions. In this acute phase, newly formed venous endothelial cells with high expression of the adhesion molecule ICAM‐1 were observed, promoting the infiltration of myeloid cells and NKT cells. From the acute to chronic phases, endothelial angiogenesis gradually decreased, accompanied by a marked increase in antigen presentation function. At 14 days post‐MCAO, an increased proportion of VCAM‐1‐expressing venous endothelial cells was observed, potentially facilitating the infiltration of T cells and a subset of neutrophils. Furthermore, we discovered that the differential changes in venous endothelial cells at different stages after MCAO may be driven by distinct differentiation and proliferation patterns regulated by different signaling pathways.

**Conclusion:**

Our study highlights that the differential expression of adhesion molecules and functional changes in endothelial cells at distinct stages after ischemic stroke may regulate the infiltration patterns of peripheral immune cells.

## Graphical abstract

In the acute phase of ischemic stroke, the proliferation of venous ECs increased, with high ICAM‐1 expression, promoting the infiltration of myeloid cells. In the chronic phase, as venous ECs repaired, their antigen presentation function was enhanced, and VCAM‐1 expression increased, facilitating the infiltration of lymphocytes.

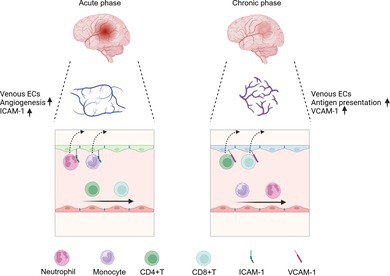

## 1 Introduction

Following an ischemic stroke, peripheral immune cells infiltrate into the brain after blood–brain barrier (BBB) dysfunction, profoundly affecting both the extent of brain damage and the subsequent repair processes [1, 2]. Numerous studies have demonstrated that the immune cell infiltration pattern following stroke is characterized by a predominance of myeloid cells in the acute phase, with lymphocytes gradually infiltrating during the subacute to chronic phases [3, 4, 5, 6, 7]. This temporal shift in infiltrated immune cell composition is believed to influence brain tissue repair and neurological function recovery following ischemic stroke [2]. However, the underlying mechanisms driving this transition remain poorly understood. Investigating whether and how the brain controls peripheral immune cell infiltration may provide promising therapeutic opportunities for the treatment of ischemic stroke.

Endothelial cells (ECs) serve as a critical interface between the blood circulation and the central nervous system (CNS), functioning as both physical and immunological barriers essential for maintaining brain homeostasis [8]. As integral components of the BBB, ECs possess tight junctions that prevent the infiltration of peripheral immune cells under homeostatic conditions, thereby preserving CNS stability. In addition to their barrier function, increasing research emphasizes the pivotal role of ECs in regulating immune responses in CNS disorders including ischemic stroke [9, 10].

Following cerebral ischemia, ECs upregulate the expression of adhesion molecules, enabling peripheral immune cells to tightly adhere to the endothelium and subsequently undergo diapedesis [11]. Notably, immune cell infiltration is a multi‐step sequential process, with adhesion regarded as both an essential and early step. Thus, while numerous studies have focused on changes in peripheral immune cells following stroke, growing evidence underscores the vascular endothelium as a critical therapeutic target for regulating immune cell infiltration [12, 13]. However, some animal and clinical studies using adhesion molecule blocking antibodies after ischemic stroke have not demonstrated clear neuroprotective effects [14, 15, 16, 17]. This may be due to the insufficient understanding of the changes in ECs at different stages of ischemic stroke.

It is believed that ECs, as well as the BBB, transition from disruption to repair during the acute to chronic phases of ischemic stroke [18]. However, these changes cannot fully account for the differential immune cell infiltration observed across different stages. In this study, we used single‐cell RNA sequencing (scRNA‐seq) to conduct an in‐depth exploration of ECs and peripheral immune cells during the acute to chronic phases following MCAO. Our findings reveal and validate that ECs play a ‘gatekeeper’ role in regulating immune cell infiltration through adaptive changes in subpopulation composition and functional states at different stages. These results further expand the potential for translational applications in modulating brain immune responses at different time points.

## 2 Results

### 2.1 Peripheral Immune Cell Infiltration Functions Exhibit Limited Changes Following Ischemic Stroke

**FIGURE 1:**
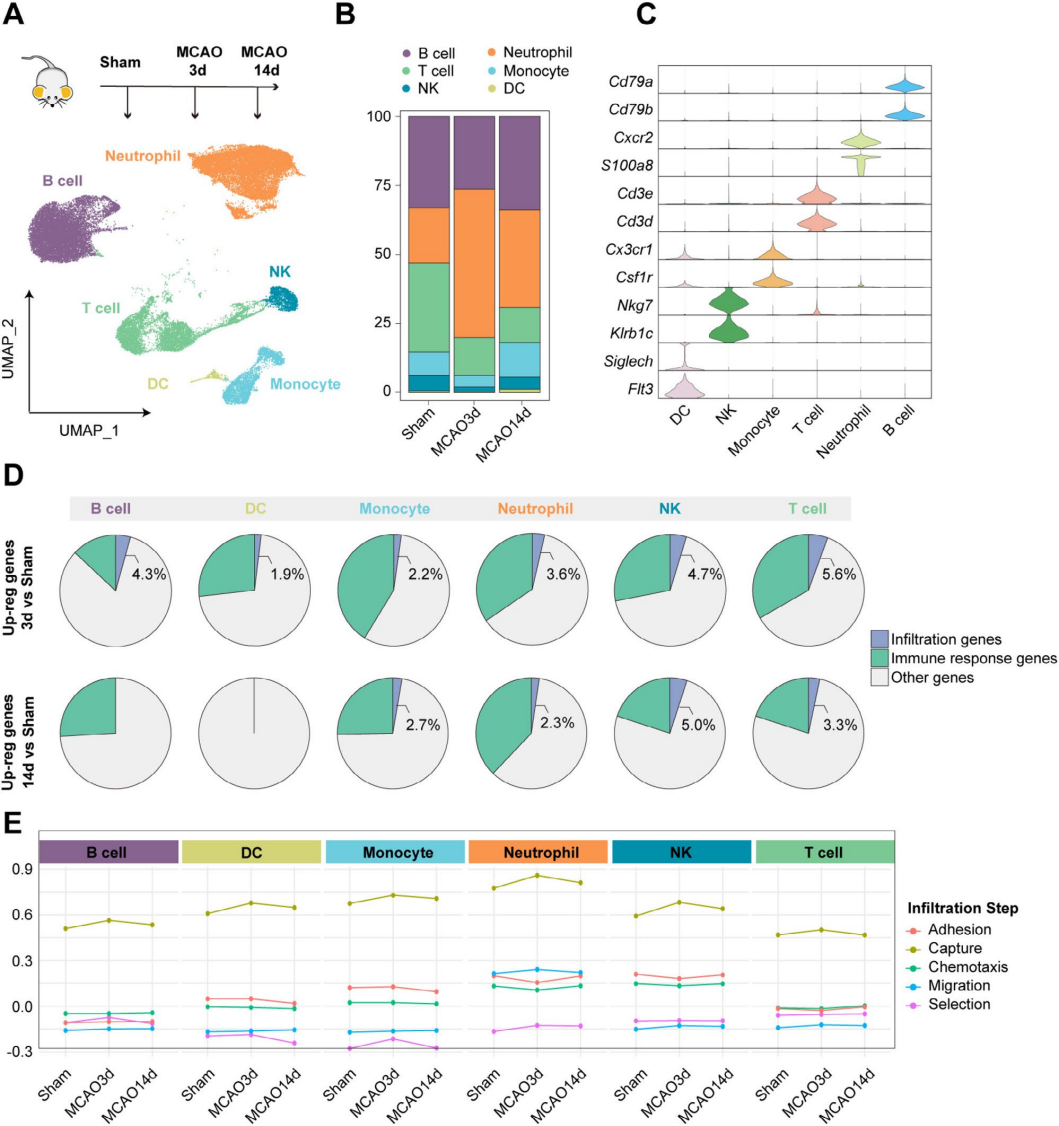
Changes in Peripheral Immune Cell Infiltration Function at Different Time Points Post‐MCAO. (A) Experimental design for scRNA‐seq of peripheral immune cells from Sham and MCAO mice (upper panel) and UMAP projection of peripheral immune cells (lower panel). *n* = 2 biological replicates per group. (B) Stacked bar plot showing the proportion of peripheral immune cell clusters in different groups. (C) Violin plot of marker gene expression for the six peripheral immune cell clusters. (D) Proportion of infiltration‐related genes, non‐infiltration‐related immune genes, and other genes among the upregulated genes in peripheral immune cells at 3 and 14 days post‐MCAO, compared to the Sham group. (E) Line chart showing functional differences in infiltration steps among the six peripheral immune cell subsets and between different groups.

To investigate whether the distinct infiltration patterns of immune cells are driven by intrinsic changes within these cells at different stages after ischemic stroke, we isolated immune cells from the peripheral circulation of mice at 3 and 14 days post‐MCAO, as well as from sham mice, and performed scRNA‐seq analysis (Figure 1A). After quality control, 44,273 cells were subjected to downstream analysis. Based on the expression of marker genes, the cells were unsupervisedly clustered into six major populations: B cells (*Cd79a*, *Cd79b*), neutrophils (*Cxcr2*, *S100a8*), T cells (*Cd3e*, *Cd3d*), monocytes (*Cx3cr1*, *Csf1r*), natural killer cells (NK, *Nkg7*, *Klrb1c*), and dendritic cells (DC, *Siglech*, *Flt3*) (Figure 1A,C, Figure S1A). Regarding changes in cell abundance, compared with the sham group, peripheral blood from mice at 3 days post‐MCAO exhibited an increased proportion of neutrophils, while the most prominent reduction among other immune cell types was observed in T cells. At 14 days post‐MCAO, the proportion of neutrophils declined, although it remained elevated relative to the sham group. Other immune cell populations generally returned to near‐baseline levels, except for T cells, whose proportions showed a decreasing trend (Figure 1A,B). These temporal changes in peripheral neutrophils and T cells were further validated by flow cytometry (Figure S1B).

We next examined whether the infiltration capacity of peripheral immune cells was altered following stroke. We observed that the proportion of upregulated infiltration‐related genes (Table S1) among the total upregulated genes in each cell cluster at 3 and 14 days post‐MCAO, compared to the sham group, was relatively low. In contrast, the proportion of immune response‐related genes not associated with infiltration was generally higher, except in DC cells at 14 days post‐MCAO (Figure 1D). Similar changes were also observed in MCAO 14 days compared to MCAO 3 days (Figure S2A), suggesting that the infiltration capacity of peripheral immune cells was not markedly enhanced at either time point.

We further explored the expression patterns of genes associated with different steps of the infiltration process, including adhesion, capture, chemotaxis, migration, and selection (Table S1). Myeloid cells, such as neutrophils and monocytes, exhibited higher expression of genes involved in capture, whereas lymphocytes showed relatively low expression of these genes. Neutrophils also demonstrated robust migration abilities. Genes involved in adhesion and chemotaxis were more highly expressed in neutrophils and NK cells compared to other populations, while T cells exhibited elevated expression of genes related to selection (Figure 1E). However, within each cell type, gene expression levels related to infiltration remained largely comparable across the sham, MCAO 3 days, and MCAO 14 days groups (Figure 1E, Figure S2B). By analyzing the expression of infiltration‐related molecules on peripheral immune cells, we found that although specific alterations were observed—such as altered expression of CXCR2 and CD62L on neutrophils, CCR2 and CD11b on monocytes, and CD62L on T and B cells at different time points—the overall expression of adhesion, chemotaxis, and selection molecules remained relatively stable across most immune cell types (Figure S2C). These findings suggest that although immune cells displayed a certain degree of activation after ischemic stroke, there might be no substantial changes in infiltration‐related gene expression. This result indicates that the observed shift from myeloid‐dominant infiltration in the acute phase to lymphoid‐dominant infiltration in the chronic phase may not be driven by intrinsic changes in the infiltration capacity of peripheral immune cells.

### 2.2 Increased Proportion of Venous ECs With Enhanced Angiogenesis and Adhesion Function at 3 Days Post‐MCAO

**FIGURE 2:**
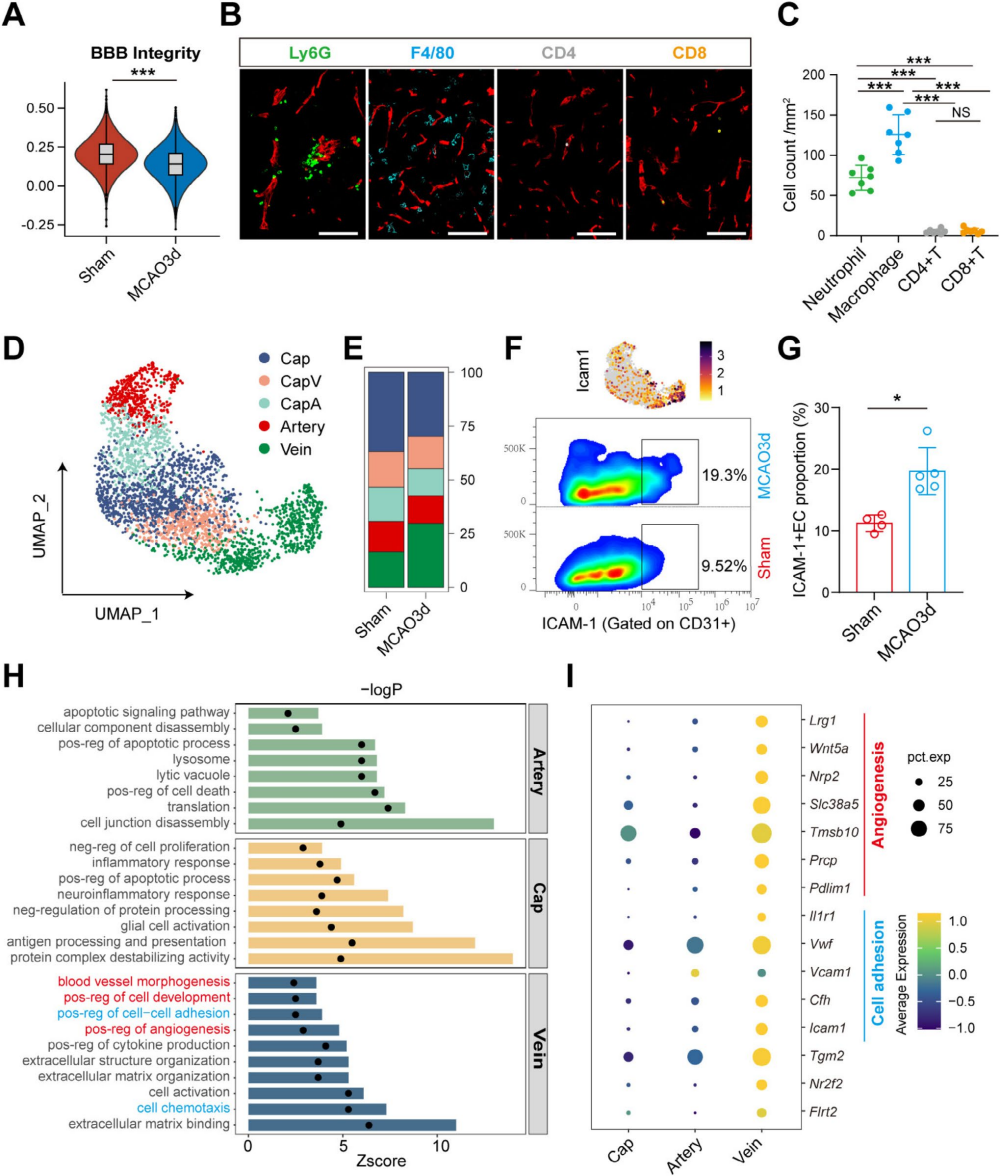
Increased Population of Venous ECs at 3 Days Post‐MCAO, with Enhanced Angiogenesis and Cell Adhesion Capabilities. (A) Violin plot showing BBB integrity in ECs between Sham and MCAO 3‐day groups. ***p value < 0.001. Bonferroni‐corrected Wilcoxon rank sum test. (B) Representative images of immunofluorescence staining of different immune cells and ECs (red) at 3 days post‐MCAO. Scale bar: 100 μm. (C) Differences in the number of various peripheral immune cells infiltrating into the brain at 3 days post‐MCAO (cells per mm^2^). ***p value < 0.0001, NS means not significant. One‐way ANOVA. *n* = 7 per group. (D) UMAP plot of ECs from sham and MCAO 3‐day mice. (E) Bar plots showing the proportions of the different ECs subsets. (F) Feature plot showing the expression of *Icam1* predominantly in veins (upper panel), and the difference in the proportion of ICAM‐1 + ECs between Sham and MCAO 3‐day groups (lower panel). (G) Flow cytometry results indicating a significant increase in the proportion of ICAM‐1 + ECs in the MCAO 3‐day group compared to the Sham group. *p value < 0.05. Mann–Whitney test. *n* = 4–5 per group. (H) Enrichment analysis of characteristic genes in three types of ECs (arteries, veins, and capillaries) at MCAO 3 days. Bars represent *z*‐score values, and dots represent significance (−log10[adjusted *p* value]). (I) Dot plot displaying venous marker genes related to angiogenesis and adhesion.

Given the limited changes observed in immune cell infiltration functions, and considering that ECs and their tight junctions act as critical barriers to immune cell entry into the brain, we performed scRNA‐seq on brain ECs from Sham and MCAO 3‐day mice for further analysis. We found that compared to the Sham group, brain ECs in MCAO 3‐day mice exhibited overall reduced BBB integrity, suggesting that the tight junctions of ECs might be disrupted during the acute phase after stroke (Figure 2A). Correspondingly, at 3 days post‐MCAO, there was infiltration of a significant number of macrophages and neutrophils into the brain. However, BBB disruption was not accompanied by an obvious infiltration of T cells during the acute phase (Figure 2B,C). This suggests that the disruption of the BBB does not lead to the unselective infiltration of immune cells into the brain.

In view of this, we further performed cluster analysis on brain ECs from Sham and MCAO 3‐day mice to analyze the changes in cell subpopulations. Based on the characteristic genes (Figure S3A), ECs were divided into capillaries (Cap), capillary venous (CapV), capillary arteries (CapA), arteries, and veins (Figure 2D). We observed that the composition of brain ECs in MCAO 3‐day mice exhibited significant changes compared to Sham mice, with a marked increase in the proportion of venous ECs (Figure 2E). *Icam1*, an adhesion molecule, is characteristically expressed on venous ECs (Figure 2F). We then used ICAM‐1 as a marker for venous ECs, and the flow cytometry results further validated that MCAO 3‐day mice had a higher proportion of ICAM‐1 + ECs in the brain compared to Sham mice (Figure 2F,G, Figure S3B).

Notably, at 3 days post‐MCAO, arteries, veins, and capillaries (including Cap, CapV, and CapA) exhibited differential function. During the acute phase, arterial ECs primarily exhibited functions related to cell death and cell junction disassembly, while capillary ECs were predominantly involved in the neuroinflammatory response. Venous ECs, on the other hand, displayed significantly higher enrichment in angiogenesis, vessel development, and regulation of cell adhesion compared to the other EC types (Figure 2H). Several genes associated with angiogenesis, such as *Lrg1*, *Wnt5a*, *Nrp2*, *Slc38a5*, *Tmsb10*, *Prcp*, and *Pdlim1*, as well as genes related to cell adhesion, including *Il1r1*, *Vwf*, *Cfh*, and *Icam1*, exhibited higher expression levels and expression ratios in venous ECs compared to other EC clusters (Figure 2I). Additionally, MCAO 3‐day mice showed a greater number of upregulated genes in venous ECs than in other ECs when compared to the Sham group (Figure S3C). These results suggest that the brain endothelium rapidly changes during the acute phase following MCAO, with an increase in the number of venous ECs associated with angiogenesis and cell adhesion.

### 2.3 Marked Angiogenesis and Increased ICAM‐1 Expression of Venous ECs, Correlated With Infiltration of Myeloid and NKT Cells During the Acute Phase of MCAO

**FIGURE 3:**
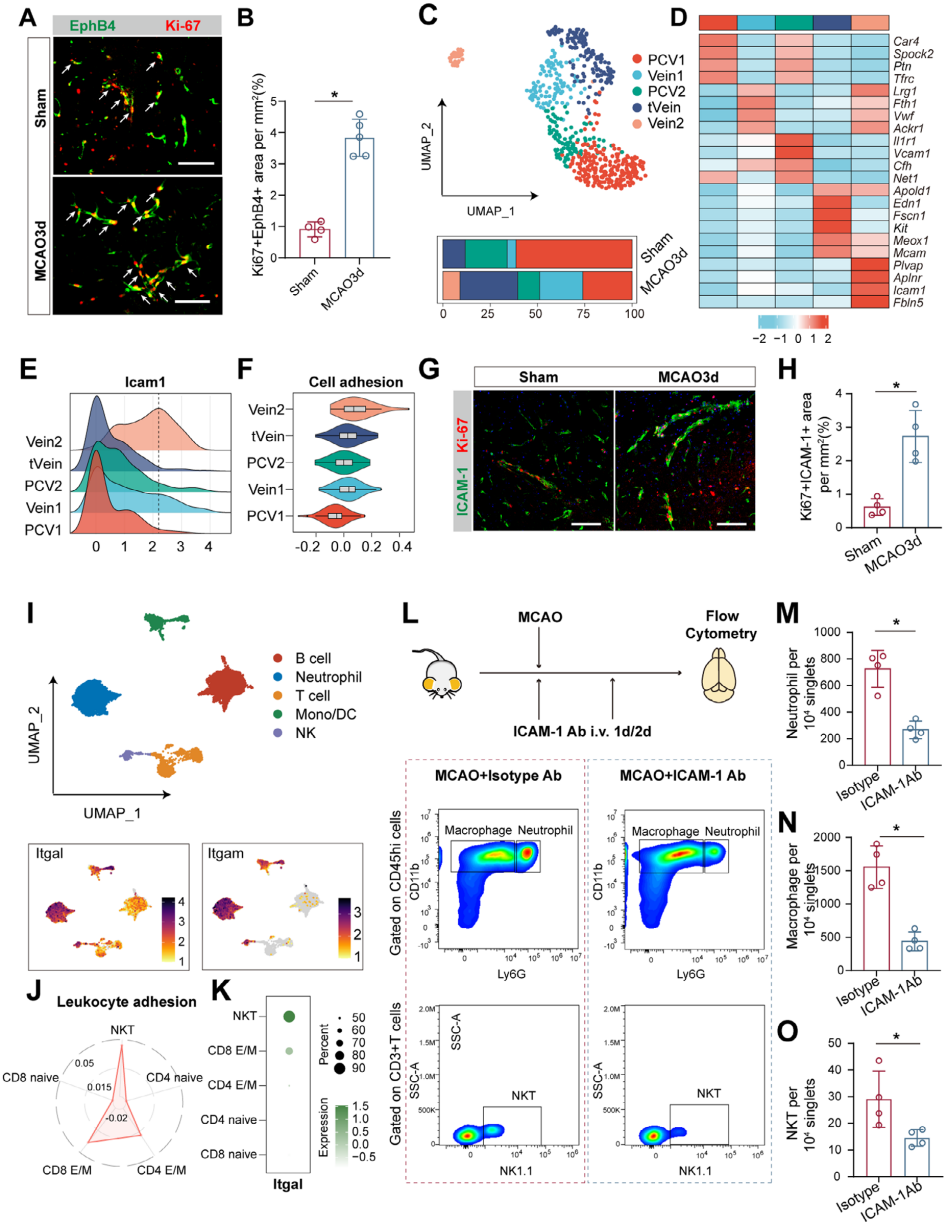
Increased expression of ICAM‐1 on newly formed venous ECs at 3 days post‐MCAO promotes the infiltration of peripheral myeloid cells and NKT cells. (A) Representative images of immunofluorescence staining for EphB4 (green) and Ki‐67 (red) in Sham and MCAO 3‐day mice. Scale bar: 100 μm; (B) significant increase in the coverage area of Ki67 + EphB4 + ECs in the brains of MCAO 3‐day mice compared to Sham mice. *p value < 0.05. Mann–Whitney test. *n* = 4–5 per group. (C) UMAP plot of venous ECs from Sham and MCAO 3‐day mice (upper panel) and the proportions of different cell subsets (lower panel). (D) Heatmap showing the marker gene expression for each venous ECs subpopulation. (E) Ridge plot displaying the expression of *Icam1* in each venous ECs subpopulation. (F) Violin plot showing the differences in cell adhesion functions among the venous EC subpopulations. (G) representative images of immunofluorescence staining for ICAM‐1 (green) and Ki‐67 (red) in Sham and MCAO 3‐day mice. Scale bar: 100 μm; (H) significant increase in the coverage area of Ki67 + ICAM‐1 + ECs in the brains of MCAO 3‐day mice compared to Sham mice. *p value < 0.05. Mann–Whitney test. *n* = 4 per group. (I) UMAP plot of peripheral immune cells from MCAO 3‐day mice (upper panel); feature plot showing the expression of *Itgal* and *Itgam* in MCAO 3‐day peripheral immune cells (lower panel). (J) Radar plot showing the differences in cell adhesion function among different peripheral T cell subsets at 3 days post‐MCAO. (K) Dot plot displaying the expression of *Itgal* in different peripheral T cell subsets at 3 days post‐MCAO. (L) Flow cytometry experimental workflow to assess the impact of ICAM‐1 on immune cell infiltration at 3 days post‐MCAO (upper panel), and flow cytometry proportion differences between the isotype and ICAM‐1 block groups (lower panel). ICAM‐1 blockade leads to a significant reduction in the infiltration of neutrophils (M), macrophages (N), and NKT cells (O) in the brain. *p value < 0.05. Mann–Whitney test. *n* = 4 per group.

We further analyzed the changes in venous ECs during the acute phase of MCAO. Compared to Sham brain venous ECs, those in MCAO 3‐day mice showed upregulated expression of angiogenesis‐related genes such as *Spp1*, *Lrg1*, *Egr1*, and *Atf3* (Figure S4A), suggesting a further increase in angiogenesis in venous ECs during the acute phase (Figure S4B). EphB4, which is associated with venous endothelial proliferation, was used as a marker to identify proliferating venous ECs [19, 20]. We found a significant increase in the coverage area of EphB4 + Ki67 + ECs in MCAO 3‐day mice compared to Sham mice (Figure 3A,B).

We further analyzed the subpopulation composition of venous ECs. Based on gene expression profiles, venous ECs were classified into five clusters: Vein1, Vein2, post‐capillary venule 1 (PCV1), post‐capillary venule 2 (PCV2), and a cluster with high expression of tip cell‐related genes (tVein) (Figure 3C,D, Figure S4C). In terms of quantity, at 3 days post‐MCAO, the proportions of PCV1 and PCV2 decreased, with PCV2 showing high expression of the adhesion molecule gene *Vcam1*. In contrast, the proportions of tVein, Vein1, and Vein2 increased (Figure 3C,D). Approximately 8% of brain venous ECs of MCAO 3‐day mice represent the Vein2 subpopulation, which is distinguished by high expression of genes such as *Plvap*, *Aplnr*, *Fbln5*, and *Icam1* (Figure 3C,D). Notably, Vein2 was absent in the venous ECs of Sham mice, suggesting that it may be a venous subpopulation unique to the acute phase of MCAO (Figure 3C). Among the cell clusters that increased at 3 days post‐MCAO, Vein2, and tVein exhibited higher angiogenesis capabilities compared to other venous ECs, while showing lower BBB integrity (Figure S4D,E). This suggests that these newly formed venous ECs might not have established a complete barrier.

At 3 days post‐MCAO, the adhesion molecule gene *Icam1* was most highly expressed in Vein2, followed by Vein1. Correspondingly, Vein2 exhibited stronger cell adhesion functions, with Vein1 showing a slightly lower level (Figure 3E,F). Given that Vein2 shows stronger angiogenic potential, we examined the expression of Ki‐67 and ICAM‐1 in the brain. The results showed that compared to Sham mice, there was a significant increase in the coverage of Ki‐67 + ICAM‐1 + ECs in the brains of MCAO 3‐day mice (Figure 3G,H). This indicates that the newly formed Vein2 at 3 days post‐MCAO highly expresses ICAM‐1. Consistently, ICAM‐1 expression at Day 3 post‐MCAO was primarily observed in EphB4 + ECs (Figure S4F).

To assess the impact of the increase in ICAM‐1 + venous ECs on immune cell infiltration, we analyzed the expression of ICAM‐1 receptors on peripheral blood immune cells at 3 days post‐MCAO (Figure 3I). The molecules encoded by *Itgal* and *Itgam* are the α subunits of LFA‐1 and integrin αMβ2, respectively, and serve as key binding sites for ICAM‐1 [21, 22]. We found that *Itgal* and *Itgam* were primarily expressed on neutrophils, monocytes, and NK cells (Figure 3I). Additionally, we also observed that among the various T cell subsets at 3 days post‐MCAO, NKT cells exhibited higher levels of adhesion ability, which may be related to their high expression of the *Itgal* gene (Figure 3J,K). This suggests that the adhesion process related to ICAM‐1 may play a key role in the infiltration of neutrophils, monocytes, NK cells, and NKT cells during the acute phase. To validate this process, we intravenously injected an ICAM‐1 blocking antibody post‐MCAO and then assessed the number of infiltrating cells into the brain at 3 days post‐MCAO (Figure 3L). The results showed that after ICAM‐1 blocking, the number of infiltrated neutrophils, macrophages, and NKT cells in the brain decreased to varying extents (Figure 3M–O), while no significant difference was observed in NK cells (Figure S4G). These results suggest that newly formed venous ECs that highly express ICAM‐1 may be involved in the immune cell infiltration process during the acute phase of MCAO.

### 2.4 ECs Changes During the Chronic Phase After MCAO

**FIGURE 4:**
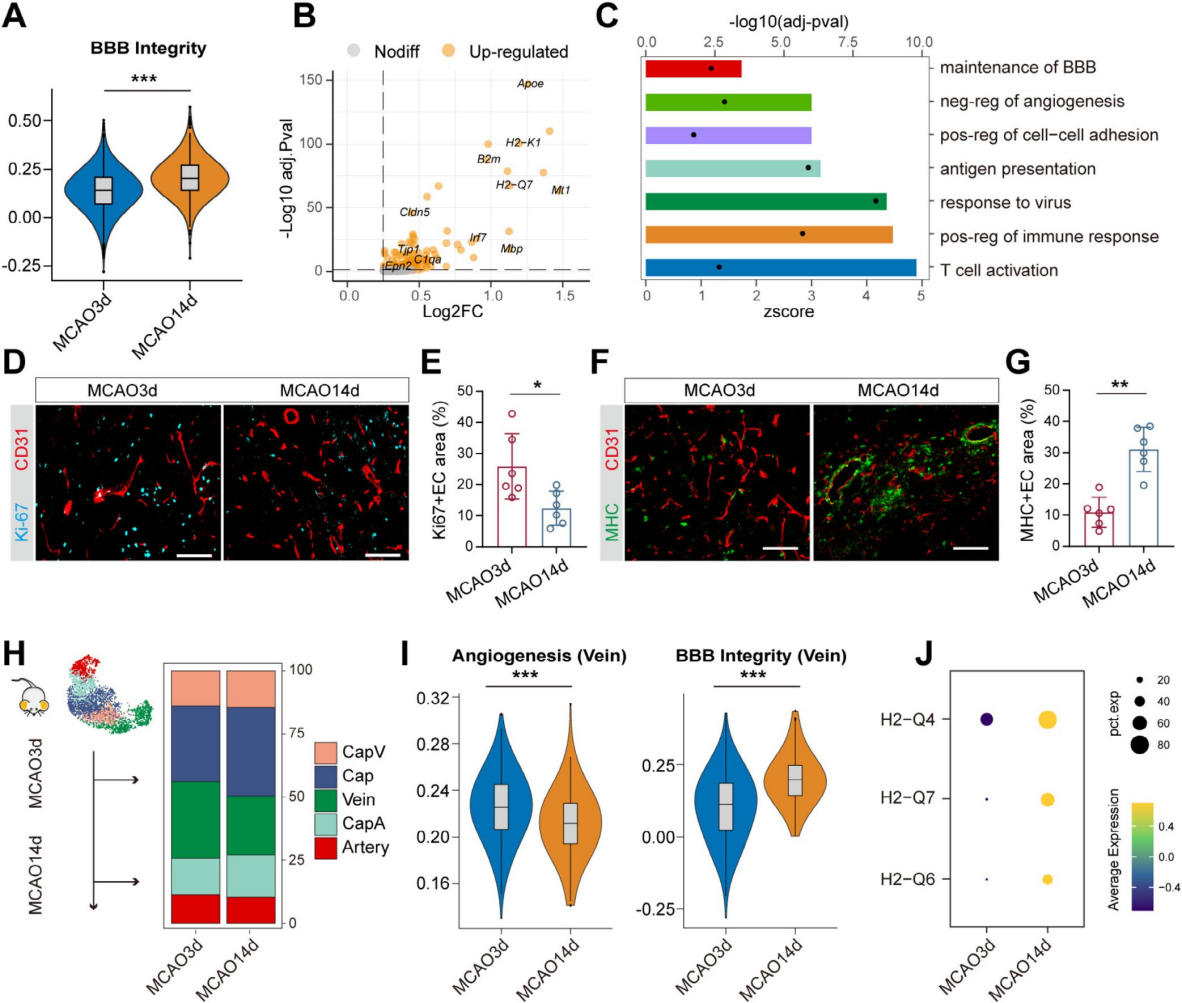
Reduced EC proliferation and increased antigen‐presenting function at 14 days post‐MCAO, predominantly in venous ECs. (A) Violin plot showing the BBB integrity of ECs in the MCAO 3‐day and MCAO 14‐day mice. ***p value < 0.001. Bonferroni‐corrected Wilcoxon rank sum test. (B) Scatter plot showing the upregulated genes (orange) with a log2(fold change) > 0.25 in the ECs of the MCAO 14‐day group compared to the MCAO 3‐day group, with log2(fold change) along the x‐axis and −log10(adjusted *p* value) along the y‐axis. (C) Bar plot displaying the pathways enriched by upregulated genes in ECs of the MCAO 14‐day group compared to the MCAO 3‐day group. Bars represent z‐score values, and dots represent −log10(adjusted *p* value). (D) Representative images of immunofluorescence staining for Ki‐67 (blue) and CD31 (red) in MCAO 3‐day and MCAO 14‐day mice. Scale bar: 100 μm. and (E) comparison between the two groups. *p value < 0.05. Mann–Whitney test. *n* = 6 per group. (F) Representative images of immunofluorescence staining for MHC (green) and CD31 (red) in MCAO 3‐day and MCAO 14‐day mice. Scale bar: 100 μm. and (G) comparison between the two groups. **p value < 0.01. Mann–Whitney test. *n* = 6 per group. (H) Stacked bar plot showing the proportion of EC clusters in MCAO 3‐day and MCAO 14‐day mice. (I) Violin plot showing the angiogenesis and BBB integrity of venous ECs in the MCAO 3‐day and MCAO 14‐day mice. ***p value < 0.001. Bonferroni‐corrected Wilcoxon rank sum test. (J) Dot plot showing the differential expression of antigen presentation‐related molecules in venous ECs between the MCAO 3‐day and MCAO 14‐day mice.

At 14 days post‐MCAO, tissue repair processes had been initiated [23], with a progressive decrease in infarct volume (Figure S5A). To investigate the role of ECs during this chronic phase after MCAO, we first compared the BBB integrity of ECs at 3 days and 14 days post‐MCAO. The results showed that by 14 days, the overall integrity of the endothelial barrier had gradually been restored (Figure 4A). However, we observed a significant infiltration of T lymphocytes into the brains of MCAO mice at 14 days post‐stroke, along with the presence of cells traditionally thought to primarily infiltrate during the acute phase, such as neutrophils and macrophages (Figure S5B,C). Since mature neutrophils are short‐lived cells that cannot proliferate further, their presence is more likely to be attributed to peripheral infiltration. These results suggest that although the BBB was in the process of being repaired during the chronic phase, immune cell infiltration persisted. We compared the upregulated genes in brain ECs of MCAO 14‐day mice with those in MCAO 3‐day mice and found that the former exhibited higher levels of gene expression related to antigen presentation and T cell activation (Figure 4B,C). In addition, at 14 days post‐MCAO, EC adhesion functions were upregulated, while angiogenesis‐related genes were downregulated (Figure 4B,C, Figure S5D). Correspondingly, we validated that compared to 3 days post‐MCAO, there was a significant decrease in Ki67 + ECs in the brains of mice at 14 days post‐MCAO, while MHC + ECs significantly increased (Figure 4D–G).

Further analysis revealed that in contrast to the increase in venous ECs during the acute phase, the number of venous ECs within the brains of mice at the chronic phase decreased (Figure 4H). Consistently, compared to MCAO 3 days, we observed a reduction in the angiogenic capacity of venous ECs at Day 14 post‐MCAO, along with an increase in BBB integrity (Figure 4I). Functional enrichment analysis showed that at 14 days post‐MCAO, arterial, capillary, and venous ECs showed varying degrees of immune regulation. Notably, venous ECs expressed higher levels of antigen presentation‐related functions compared to other ECs types (Figure S5E,F). Additionally, we observed that the expression of antigen presentation molecules in venous ECs was higher at 14 days post‐MCAO compared to 3 days post‐MCAO (Figure 4J). These results suggest that although the proportion of venous ECs within the brain decreases at 14 days post‐MCAO, they may still serve as an important pathway for immune cell infiltration.

### 2.5 Increased Expression of VCAM‐1 on Venous ECs and Its Role in T Cell Infiltration During the Chronic Phase of MCAO

**FIGURE 5:**
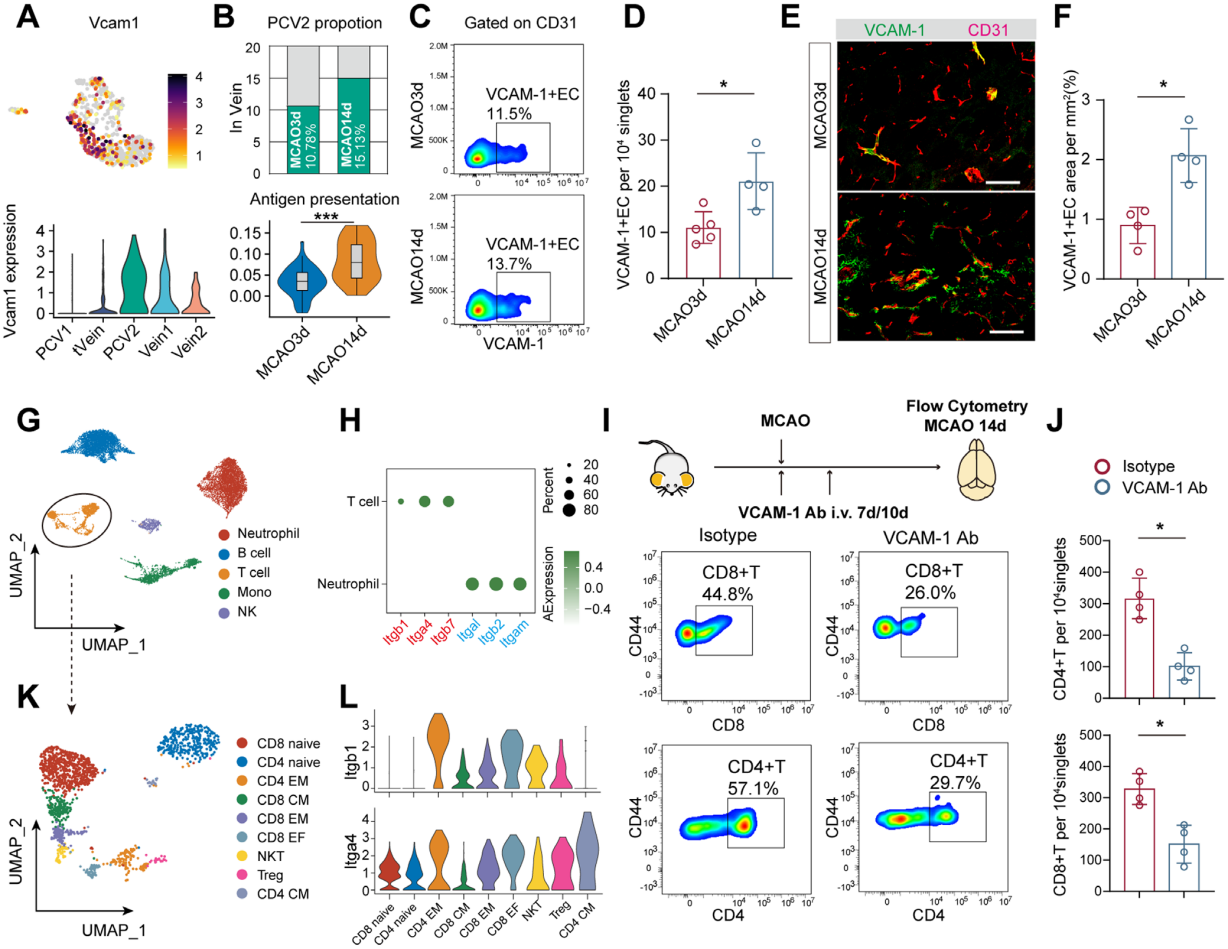
At 14 days post‐MCAO, an increase in the PCV2 subpopulation, characterized by high VCAM‐1 expression, promotes T cell infiltration. (A) Feature plot (upper) and violin plot (lower) showing that *Vcam1* is predominantly expressed in the PCV2 subpopulation. (B) Comparison of the proportion of PCV2 in venous ECs (upper) and violin plot showing the antigen presentation function differences (lower) between the MCAO 3‐day and MCAO 14‐day mice. ***p value < 0.001. Bonferroni‐corrected Wilcoxon rank sum test. (C) Flow cytometry analysis comparing the proportion of VCAM‐1 + ECs at 3 days and 14 days post‐MCAO and the results (D) showing that the MCAO 14‐day group has a higher number of VCAM‐1 + ECs in the brain compared to the MCAO 3‐day group. *p value < 0.05. Mann–Whitney test. *n* = 4–5 per group. (E) Immunofluorescence staining detecting the coverage area of VCAM‐1 (green) + CD31+ (red) ECs in the brains of MCAO 3‐day and MCAO 14‐day mice and (F) comparison between the two groups. *p value < 0.05. Mann–Whitney test. *n* = 4 per group. (G) UMAP plot of peripheral immune cells from MCAO 14‐day mice. (H) Dot plot displaying the expression of adhesion molecule receptor genes in peripheral T cells and neutrophils from MCAO 14‐day mice (red for VCAM‐1 receptor genes; blue for ICAM‐1 receptor genes). (I) Flow cytometry experimental workflow to assess the impact of VCAM‐1 on immune cell infiltration at 14 days post‐MCAO (upper panel), and flow cytometry proportion differences between the Isotype and VCAM‐1 block groups (lower panel). (J) VCAM‐1 blockade leads to a reduction in the infiltration of CD4 + T cells and CD8 + T cells. *p value < 0.05. Mann–Whitney test. *n* = 4 per group. (K) UMAP plot of peripheral T cells from MCAO 14‐day mice. (L) Violin plot showing higher expression of genes encoding the two subunits of VLA‐4 (*Itgb1* and *Itga4*) in CD4 EM and CD8 EF clusters. CM refers to central memory T cells, EM refers to effector memory T cells, and EF refers to effector T cells.

To further analyze the impact of venous changes on immune cell infiltration during the chronic phase of MCAO, we examined the expression of adhesion molecules in venous subpopulations. We found that the Vein2 cluster, which highly expressed *Icam1* at 3 days post‐MCAO, exhibited a markedly lower proportion at Day 14 (Figure S6A). In contrast, the proportion of PCV2 was increased, characterized by high *Vcam1* expression and elevated antigen‐presenting capacity (Figure 5A,B, Figure S6A,B). We then compared the number of VCAM‐1 + ECs between MCAO 3‐day and 14‐day mice and found that MCAO 14‐day mice had a significantly higher number of VCAM‐1 + ECs in the infarcted hemisphere (Figure 5C,D). Similarly, the coverage area of VCAM‐1 + ECs at 14 days post‐MCAO was also significantly larger (Figure 5E,F).

We further analyzed the expression of adhesion molecule receptors in peripheral immune cells, particularly T cells and neutrophils, which represent classical and non‐classical immune cell populations infiltrating the brain during the chronic phase post‐MCAO (Figure 5G). It was shown that T cells predominantly expressed genes encoding the VCAM‐1 receptor (*Itgb1*, *Itga4*, *Itgb7*) while neutrophils showed high expression of genes encoding the ICAM‐1 receptor (*Itgal*, *Itgb2*, *Itgam*) (Figure 5H). These findings suggest that VCAM‐1 might be crucial for the adhesion and infiltration of T cells. Therefore, we intravenously injected a VCAM‐1 blocking antibody into mice on Days 7 and 10 post‐MCAO and assessed the number of T cells infiltrating into the brain at Day 14 by flow cytometry (Figure 5I). The results showed that compared to control mice, VCAM‐1 blockade led to a significant reduction in the number of infiltrating CD4 + and CD8 + T cells (Figure 5J). Additionally, further analysis of T cell subsets revealed that *Itgb1* and *Itga4* were highly expressed in CD4 EM (CD4 effector memory) and CD8 EF (CD8 effector) cells (Figure 5K,L), suggesting that these subsets are more likely to infiltrate the brain at 14 days post‐MCAO through VCAM‐1 adhesion molecules. Since a group of neutrophils remained at 14 days post‐MCAO, we also examined the effect of VCAM‐1 blockade on their infiltration. The results showed that following VCAM‐1 blockade, the number of infiltrated neutrophils decreased, although the reduction was modest (Figure S6C,D). Further analysis of peripheral neutrophil subsets revealed that a subset of neutrophils, specifically Neu4, exhibited high *Itga4* expression, suggesting that these cells may infiltrate the brain via VCAM‐1 (Figure S6E,F).

### 2.6 Distinct Transcriptional Regulation Drives Differential Differentiation and Proliferation Patterns of Venous ECs During the Acute and Chronic Phases After MCAO

**FIGURE 6:**
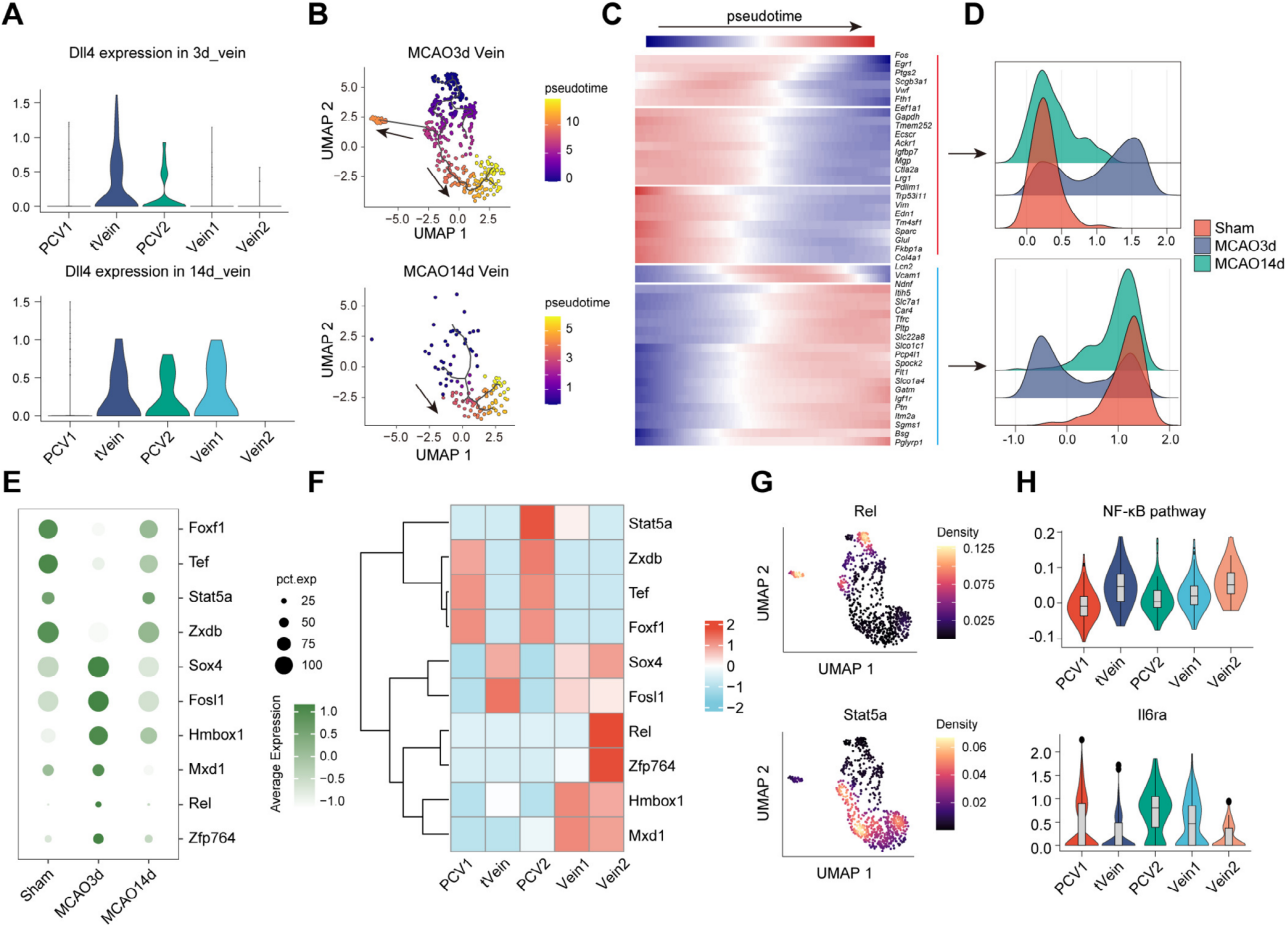
Distinct transcriptional programs underlie the differential differentiation and proliferation of venous ECs during the acute and chronic phases after MCAO. (A) Violin plot showing differential expression of *Dll4* in venous ECs subpopulations at MCAO 3 days and MCAO 14 days. (B) Differentiation trajectory of venous ECs at 3 days and 14 days post‐MCAO. (C) Heatmap showing gene expression differences in venous ECs at different pseudotime stages. (D) Ridge plot illustrating that genes expressed in early pseudotime are predominantly upregulated in venous ECs at MCAO 3 days (upper panel), while genes expressed in later pseudotime are predominantly upregulated in venous ECs from Sham and MCAO 14‐day mice. (E) Dot plot depicting differential transcriptional regulatory activity in venous ECs from Sham, MCAO day 3, and MCAO day 14 groups. (F) Heatmap showing differences in transcription factor regulatory activity across venous ECs subpopulations. (G) Density plot displaying the expression distribution of the transcription factors *Rel* and *Stat5a* and their target genes in venous ECs. (H) Violin plot showing higher expression of NF‐κB pathway‐related genes in Vein2 and tVein, and higher expression of *Il6ra* in PCV.

To further explore the potential reasons behind the changes in venous ECs at different pathological stages following ischemic stroke, we analyzed the differentiation of venous ECs. By using Cytotrace [24], we observed that both at 3 and 14 days post‐MCAO, the overall differentiation level of cells was lower in the Vein clusters, including Vein1, Vein2, and tVein, while the differentiation levels in PCV1 and PCV2 were higher (Figure S6G). From this perspective, the repair of venous ECs is more likely to proceed from the Veins to the PCVs. However, we also observed that due to the different composition of venous clusters, the starting points for venous differentiation varied between the acute and chronic phases of MCAO (Figure S6G). Sprouting of tip cells, characterized by the expression of *Dll4*, is a crucial early event in the process of angiogenesis [25]. We observed that in the brains of MCAO 3‐day mice, *Dll4* was predominantly expressed in the tVein cluster, with moderate expression in the PCV2, while at 14 days post‐MCAO, *Dll4* expression was similar in tVein, PCV2, and Vein1 (Figure 6A). Therefore, due to the differential expression of *Dll4*, the distribution of tip cell subpopulations varied between 3 and 14 days post‐MCAO, leading to distinct proliferation and differentiation processes: in the MCAO 3‐day mice, differentiation occurred from tVein to Vein2 or PCVs, while in the MCAO 14‐day mice, tVein and Vein1 differentiated into PCVs (Figure 6B).

During the above process, expression of a group of genes varied along pseudotime (Figure 6C). Specifically, genes expressed in early pseudotime were highly expressed in the venous ECs of MCAO 3‐day mice, whereas venous ECs in MCAO 14‐day mice exhibited higher expression of genes associated with the later stages of pseudotime (Figure 6D). This not only reflects the differences in the proportion of venous EC subpopulations with varying differentiation states at various time points, but also highlights the ‘rapid response’ initiated by venous ECs during the acute phase of MCAO, followed by a gradual return to steady state as they enter the later stages of repair.

Additionally, we further observed significant differences in the transcription factor activity of venous ECs across different groups. A group of transcription factors, such as *Sox4*, *Fosl1*, *Rel*, and *Zfp764*, exhibited higher regulatory activity in the venous ECs of MCAO 3‐day mice, while *Foxf1*, *Tef*, *Stat5a*, and *Zxdb* were more highly active in Sham and MCAO 14‐day mice (Figure 6E). Different venous ECs also demonstrated distinct transcription factor regulatory activities (Figure 6F). Notably, we found that *Stat5a* exhibited significantly higher regulatory activity in PCV2, whereas *Rel* and *Zfp764* showed increased regulatory activity in Vein2 (Figure 6F,G). According to previous reports, the *Rel* transcription factor is typically associated with higher NF‐κB signaling activity [26]. Consistently, we observed that genes related to the NF‐κB pathway were more highly expressed in tVein and Vein2 compared to other clusters (Figure 6H), suggesting that Rel or NF‐κB may be important regulators of venous endothelial proliferation and differentiation during the acute phase of MCAO. In contrast, although *Stat5a* was highly expressed in PCV2, we did not observe an upregulation of the JAK–STAT signaling pathway in this cluster (Figure S6H). Interestingly, we found a significant upregulation of *Il6ra* (Figure 6H). Previous literature has reported that the Il6‐Il6ra signaling pathway can regulate *Stat5* to influence angiogenesis [27, 28, 29], suggesting that this pathway may be a key target influencing the proliferation and differentiation of PCV2 at 14 days post‐MCAO.

## 3 Discussion

Previous studies have reported that in the context of stroke, ECs upregulate the expression of adhesion molecules following ischemia–reperfusion injury [30, 31], which facilitates the infiltration of peripheral immune cells into the ischemic brain tissue, thereby exacerbating the inflammatory response [10]. Despite this, ECs have long been considered ‘passive bystanders’ in these processes [9], with numerous studies focusing on how peripheral immune cells alter their function post‐injury and subsequently adhere to the ‘passive’ ECs. In this manuscript, we present evidence that the endothelium undergoes significant changes during different phases post‐MCAO, actively participating in the process of immune cell infiltration.

The temporal and spatial dynamics of peripheral immune cell infiltration following ischemic stroke have long been a focus of attention, as regulating immune responses has been proven to be a promising therapeutic strategy for mitigating secondary damage after reperfusion therapy [32, 33, 34]. In our study, peripheral immune cells from mice were isolated for scRNA‐seq analysis. The results revealed that compared to the sham group, immune cells exhibited functional alterations during both the acute and chronic phases of MCAO. However, changes in infiltration capacities after injury, as well as during the transition from the acute to chronic phases, were relatively modest. These results may suggest that peripheral immune cells retain the ability to infiltrate into the brain at different stages. In a recent study, Garcia‐Bonilla et al. performed scRNA‐seq analysis on peripheral immune cells at 2 and 14 days post‐MCAO and observed less phenotypic differentiation in circulating leukocytes compared to brain‐recruited leukocytes after stroke [4]. This highlights that regulating the process of immune cell infiltration may have greater priority and value compared to modulating their function after infiltration.

Studies have reported changes in ECs following ischemic stroke, particularly the upregulation of adhesion molecules such as ICAM‐1 and VCAM‐1 [18]. In addition, stroke‐related risk factors such as aging and elevated blood pressure have also been reported to significantly affect the expression of VCAM‐1 and ICAM‐1 in both ECs and peripheral immune cells [35, 36, 37, 38], further highlighting the importance of understanding the regulatory mechanisms underlying these molecules and developing targeted interventions in stroke treatment. However, the specific types of ECs and the pathological stages following stroke where these adhesion molecule changes occur have not been further explored. Consistent with previous reports, our study clarifies that the main site of immune cell infiltration after stroke is likely to be the venous ECs [39, 40]. In addition, we have found and validated that during the acute phase of stroke, venous ECs undergo rapid angiogenesis in response to injury. These newly formed ECs do not yet form complete tight junctions and exhibit high expression of ICAM‐1, providing a favorable environment for the infiltration of myeloid cells as well as NKT cells. In the chronic phase of stroke, we observed that as venous ECs gradually regenerate, the expression of VCAM‐1 is further upregulated, promoting the infiltration of lymphocytes, particularly T cells.

Previous studies using adhesion molecule block antibodies after ischemic stroke, which did not yield conclusive neuroprotective effects [14, 15, 16, 17], may also be related to the fact that ECs have roles beyond immune cell adhesion. Our present study found that venous ECs exhibit strong angiogenic potential during the acute phase, whereas in the chronic phase, their characteristic function is enhanced antigen presentation. Notably, during the acute phase, there are unique subpopulations of newly formed venous ECs with strong cell adhesion capabilities, which, however, are almost absent in the chronic phase. The origins and fate of these cells warrant further investigation. Another interesting finding is that at 14 days post‐MCAO, while the composition of ECs, at least in terms of quantity, is nearly comparable to that of sham mice, there is a characteristic upregulation of antigen presentation function. The antigen presentation capacity of ECs has been studied over the years, yet it remains unclear whether this function, in coordination with the high expression of VCAM‐1, contributes to the infiltration of T cells during the chronic phase of ischemic stroke. Moreover, we observed distinct transcription factor activities across venous ECs at different time points following MCAO, suggesting that the regulation of key transcription factors may modulate venous EC function throughout various stages of stroke. This, in turn, could influence immune cell recruitment and infiltration, offering a potential avenue for targeted immunomodulatory strategies during stroke progression.

This study also has some limitations. First, although biological validations were performed, the sample size for the scRNA‐seq experiments was relatively small, which may have affected the generalizability of the results. Second, we did not correlate the functional changes of ECs with vascular structural alterations post‐MCAO. Third, we did not clarify whether peripheral immune cells exert further effects on ECs upon adhesion. Lastly, although we proposed distinct differentiation patterns of venous ECs at different stages after MCAO, we did not elucidate whether these differences arise from intrinsic endothelial properties or are influenced by interactions with other cell types, such as microglia.

In summary, our study identifies and proposes that the varying expression of adhesion molecules and cellular functions in ECs at different phases after ischemic stroke may lead to the differentiated infiltration of peripheral immune cells. These results provide a foundation for future therapeutic strategies aimed at modulating brain immune responses through the regulation of ECs.

## 4 Methods

### 4.1 Animals

Young (8–10 week old) male C57BL/6 mice were purchased from Shanghai SLAC Laboratory Co. Ltd. (Shanghai, China). All animals used in the in vivo experiments were housed in temperature‐ and humidity‐controlled plastic cages under a 12‐h light/dark cycle, with ad libitum access to water and food. Animals were randomly assigned to experimental groups prior to surgery and randomly selected to undergo subgroup investigations at different survival times. Efforts were made to minimize animal suffering and reduce the number of mice utilized in the study. All experimental procedures involving animals were reviewed and approved by the Institutional Ethics Committee of the Second Affiliated Hospital, Zhejiang University School of Medicine. These procedures were conducted in strict accordance with the guidelines outlined in the National Institutes of Health Guide for the Care and Use of Laboratory Animals.

### 4.2 Murine Models of Transient Middle Cerebral Artery Occlusion

The transient middle cerebral artery occlusion (MCAO) model was induced based on previously described methods [41, 42]. Briefly, mice were deeply anesthetized with 1% pentobarbital and secured on a plastic board after confirming the absence of a response to tail pinch tests. The common carotid artery (CCA), external carotid artery (ECA), and internal carotid artery (ICA) were carefully exposed. A silicone‐coated filament was introduced into the ECA through a small incision and advanced into the ICA. The filament was gently maneuvered until resistance was felt, indicating the occlusion of the origin of the middle cerebral artery (MCA) and induction of transient cerebral ischemia. The filament remained in place for 60 min to maintain the occlusion, after which it was withdrawn to restore cerebral blood flow. The residual segment of the ECA was then ligated, and the neck incision was carefully sutured. Successful MCAO induction was confirmed using laser speckle contrast imaging, with only animals showing a ≥ 70% reduction in regional cerebral blood flow in the MCA territory considered successful models [7]. During the procedure, mice received a 30% O_2_/70% N_2_O mixture via nasal cannula to ensure adequate respiration, and body temperature was maintained at 37.0°C ± 0.5°C throughout the surgery. Mice in the sham group underwent identical surgical procedures, except for MCA occlusion. Following surgery, all mice were placed on a warming blanket until they regained consciousness. Perioperative pain management was achieved using ketoprofen (1–3 mg/kg body weight) via intraperitoneal injection. Anti‐mouse CD106 (VCAM‐1) antibody (BioXcell, BE0027) and anti‐mouse CD54 (ICAM‐1) antibody (BioXcell, BE0020‐1) were administered via intravenous injection to block cell adhesion molecules. The personnel who performed the experiments were blinded to the treatment groups at different time points post‐MCAO. The mortality rates in this study are listed in Table S3.

### 4.3 Immunofluorescence Staining

Briefly, brain slices from each mouse were selected and rinsed twice with PBS for 5 min. When required, membrane permeabilization was achieved by treating the slices with 0.5% Triton X‐100 in PBS (PBST) for 15 min at room temperature. The slices were then washed three times with 0.3% PBST for 5 min each. To block nonspecific binding, the brain sections were incubated with 5% normal donkey serum diluted in 0.3% PBST for 1 h at room temperature. Following blocking, the sections were incubated overnight at 4°C with the following primary antibodies: anti‐Ly6G (Proteintech, RB6‐8C5, 1:75), anti‐CD31 (R&D, AF3628, 1:200), anti‐EphB4 (R&D, AF446, 1:100), anti‐ICAM‐1 (ThermoFisher, 14‐0542‐82, 1:100), anti‐MHC‐II (ThermoFisher, 14–5321‐85, 1:100), anti‐VCAM‐1 (Abcam, ab134047, 1:100), anti‐F4/80 (ThermoFisher, 14‐4801‐82, 1:100), anti‐CD8a (Abcam, ab217344, 1:100), anti‐Ki67 (Abcam, ab155680, 1:100), and anti‐alpha smooth muscle actin (Abcam, ab124964, 1:100). The following day, the sections were rinsed three times with 0.3% PBST for 10 min each and incubated with the appropriate secondary antibodies conjugated with Alexa Fluor 488, 555, 594, or 647 (Invitrogen, 1:500) in a light‐protected environment at room temperature for 1 h. Afterward, the slices were washed three times with PBS and mounted onto glass slides using a mounting medium containing 4′,6‐diamidino‐2‐phenylindole (DAPI; Abcam). Coverslips were applied to finalize the preparation. Immunofluorescent images of the brain sections were acquired using a Leica TCS SP8 laser scanning confocal microscope and a Leica DM68 microscope (Leica Microsystems).

### 4.4 Flow Cytometry

The preparation of single‐cell suspensions of intracranial immune cells was carried out as previously described [7]. Briefly, mice were deeply anesthetized and transcardially perfused with 20 mL of precooled PBS. The brain was dissected, and the ischemic hemisphere was isolated. Brain tissue was dissociated into single‐cell suspensions using the Neural Tissue Dissociation Kit (T) (Miltenyi Biotec, 130‐093‐231) in combination with a gentleMACS Octo Dissociator with Heaters (Miltenyi Biotec), following the manufacturer's instructions. The homogenized samples were passed through a 70‐μm cell strainer and centrifuged at 400 × g for 10 min. The resulting pellet was resuspended in 10 mL of 30% Percoll and subjected to density gradient centrifugation using a 30%/70% Percoll gradient at 800 × g for 30 min. The intermediate layer, containing the desired cells, was carefully collected, washed with PBS, and stained with the following fluorochrome‐conjugated antibodies against surface antigens: anti‐CD45‐FITC (BD, 553079), anti‐CD45‐Pacific Blue (BioLegend, 103126), anti‐CD11b‐PE‐Cy7 (BioLegend, 101216), anti‐Ly6G‐PerCP‐Cy5.5 (BioLegend, 127617), anti‐NK1.1‐BV605 (BioLegend, 108740), anti‐CD3e‐FITC (BD, 553061), anti‐CD3e‐APC (BioLegend, 100311), anti‐CD4‐APC‐Cy7 (BD, 552051), anti‐CD8a‐APC (BD, 553035), anti‐CD19‐PE (BD, 557399), anti‐CD19‐BV785 (BioLegend, 115543), anti‐CD62L‐BUV395 (BD, 740218), anti‐CD54‐PE‐Cyanine7 (BioLegend, 116121), anti‐CD31‐BV421 (BioLegend, 102424), anti‐CD106‐APC (BioLegend, 105717), anti‐CD49d‐BV786 (BD, 740862), anti‐CD182‐BV605 (BD, 747814), anti‐CD44‐V500 (BD, 560781), anti‐CD192‐PE (BioLegend, 150609), anti‐CD195‐APC (BioLegend, 107011) and anti‐CD197‐BUV496 (BD, 741195). Staining was performed in the dark for 30 min on ice. Fluorochrome compensation was conducted using single‐stained UltraComp eBeads (Thermo Fisher Invitrogen). Flow cytometry was performed on a Beckman CytoFLEX LX flow cytometer (Beckman Coulter), and the acquired data were analyzed using FlowJo software.

### 4.5 Collection of Immune and Endothelial Cells for scRNA‐Seq

To investigate the interaction between peripheral blood immune cells and brain ECs, we collected peripheral blood and brains from sham, MCAO 3‐day, and MCAO 14‐day mice. Whole brain cells were obtained using the Adult Brain Dissociation Kit (ABDK, Miltenyi Biotec). Briefly, brain tissues were excised and enzymatically processed following the protocol provided by the manufacturer. The dissociation involved enzymatic treatment with the supplied enzyme mix and mechanical processing using a gentleMACS Dissociator (Miltenyi Biotec). The resulting cell suspension was filtered through a 70 μm cell strainer to eliminate debris and aggregates. The single‐cell suspension was washed twice with PBS and resuspended in PBS containing 0.04% BSA to prepare for downstream single‐cell sequencing. Peripheral immune cells were obtained by performing red blood cell lysis on peripheral blood. Cells were then introduced into a Chromium Single‐Cell Controller Instrument (10× Genomics). scRNA‐seq libraries were generated using the 10× Genomics recommended protocol. Libraries for scRNA‐seq were constructed following the 10× Genomics standard workflow. All libraries underwent sequencing on an Illumina NovaSeq platform with 2 × 150 paired‐end kits, conducted at Novogene.

### 4.6 Preliminary Analysis of scRNA‐Seq Data: Quality Control, Normalization, Dimensionality Reduction, and Clustering

FastQC was utilized to assess the quality metrics of the raw reads. The FASTQ files were processed using the Cell Ranger pipeline (Version 7.0.0, 10× Genomics) [43]. The raw data were aligned to the mm10 mouse reference genome to produce a filtered gene‐barcode matrix, including cellular barcodes and gene counts. All analyses were conducted in R software (Version 4.2.1, Vienna, Austria) using RStudio. Data visualization and statistical summaries were primarily generated using ggplot2 (Version 3.4.2) and ggpubr (Version 0.6.0) R packages.

Count matrices were processed and integrated using Seurat (Version 4.4.0) [44] and Harmony (Version 0.1.1) [45] R packages. Low‐quality cells were excluded, and the filtered matrix was subsequently normalized using Seurat. Data were scaled based on the top 2000 most variable genes and subjected to principal component analysis (PCA). The FindNeighbors function in Seurat was used to identify nearest neighbors for graph‐based clustering, leveraging principal components. Cell subtypes were defined using the FindClusters function in Seurat. Cell populations were visualized with Uniform Manifold Approximation and Projection (UMAP).

### 4.7 Differential Expression Analysis and Enrichment Analysis

Differentially expressed genes (DEG) were identified between clusters using the FindAllMarkers and FindMarkers functions. The Wilcoxon rank‐sum test was applied to compute *p* values, and Bonferroni correction was used to adjust P values for all genes. The adjusted *p* value < 0.05 and |log2foldchange| > 0.25 were set as the threshold for significantly differential expression. Gene Ontology (GO) enrichment analysis of DEGs [46, 47, 48] was conducted and visualized using clusterProfiler (Version 4.0.5) and enrichplot (Version 1.18.4) R packages with a significance level of *p* < 0.05 [49, 50]. Pathway enrichment scores were calculated using the AddModuleScore function in Seurat, based on functional gene sets related to BBB integrity, cell adhesion, angiogenesis, NF‐κB signaling, and JAK–STAT signaling, as curated from GO terms and the KEGG database (see Table S2 for details).

### 4.8 Transcription Factor Regulon Analysis

The analysis of the regulatory network and regulon activity was performed by pySCENIC [51]. The regulon activity (measured in AUC) was analyzed by the AUCell module of pySCENIC, and the activity of these regulons is quantified via the AUCell default threshold [52]. The differential‐expression regulon was identified by the Wilcoxon rank‐sum test in “FindAllMarkers” function in the R package Seurat. The scaled expression of regulon activity was used to generate a heatmap.

### 4.9 Pseudo‐Time Trajectory Analysis

To investigate the maturation of neutrophils, we utilized the R package Monocle (version 2.26.0) and Monocle3 (version 1.3.1) [53]. The cellular trajectory analysis was performed using the learn_graph function. The results of pseudotime analysis were visualized on the UMAP dimensional scatter plot. Pseudotime represents the evolutionary trajectory of the cells.

### 4.10 Statistical Analysis

Data are presented as the mean ± standard deviation (SD). The Wilcoxon rank‐sum test was applied for statistical analysis of the scRNA‐seq datasets. The Mann–Whitney *U* test was applied for comparisons between two groups when the data were not normally distributed or sample sizes were small. One‐way analysis of variance (ANOVA) was employed to assess differences in means across multiple groups. All statistical analyses were performed using R or GraphPad Prism (Version 9.5.0), with a *p* value < 0.05 considered statistically significant.

## Author Contributions

Xuejiao Dai, Xiaotao Zhang, and Jiarui Chen contributed equally to this work. Jianan Lu and Ligen Shi designed the research. Qia Zhang and Huaming Li performed the experiments. An Ping and Yuchen Liu contributed to bioinformatics and statistical analysis. Jianan Lu wrote the original manuscript. Ligen Shi, Jingyi Zhou, and Jianmin Zhang reviewed and polished the manuscript.

## Conflicts of Interest

The authors declare no conflicts of interest.

## Supporting information


Figure S1.

Table S1.

## Data Availability

The scRNA‐seq data generated in this study are available from the corresponding author upon reasonable request. Source data are provided with this paper (Table S4).
