# Supplementary material for: Endothelial Changes at Different Stages After Ischemic Stroke Contribute to the Regulation of Immune Cell Infiltration
Source: CNS Neurosci Ther. 2025 May 27;31(5):e70456. doi: 10.1111/cns.70456 (PMC12107272; doi:10.1111/cns.70456)
Supplement: Supplementary file 1 — Figure S1. [file CNS-31-e70456-s001.docx]

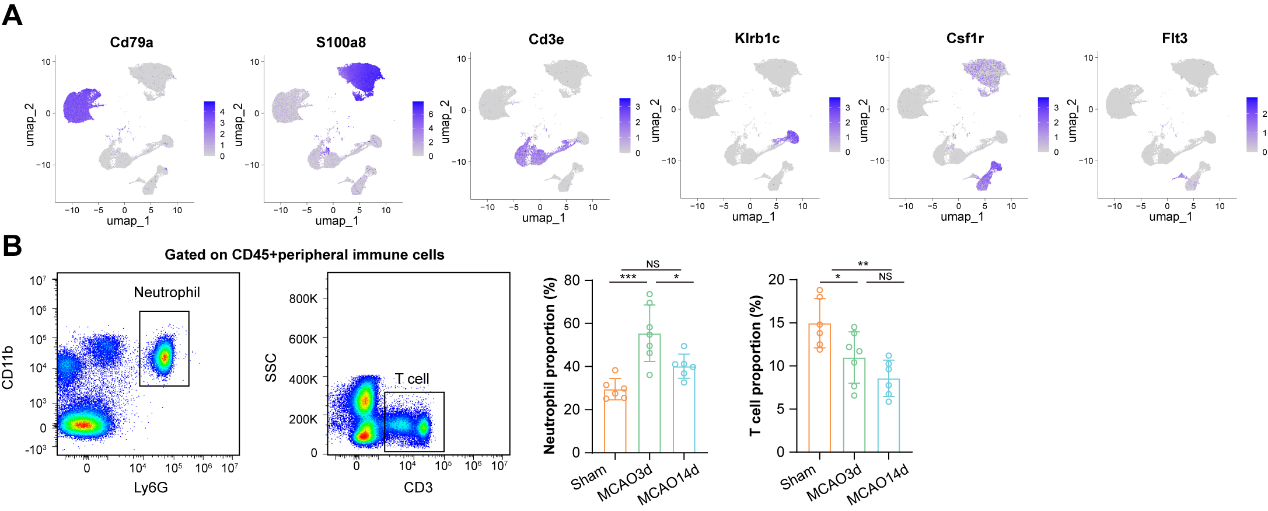


**Figure S1.**

**A)** Feature plot showing the expression of marker genes in the six peripheral immune cell clusters.

**B)** Flow cytometry gating strategy for peripheral blood neutrophils and T cells, and comparison of cell counts across groups. *P-value < 0.05, **P-value < 0.01, ***P-value < 0.001, NS means not significant. ONE-Way ANOVA. n = 6-7 per group.


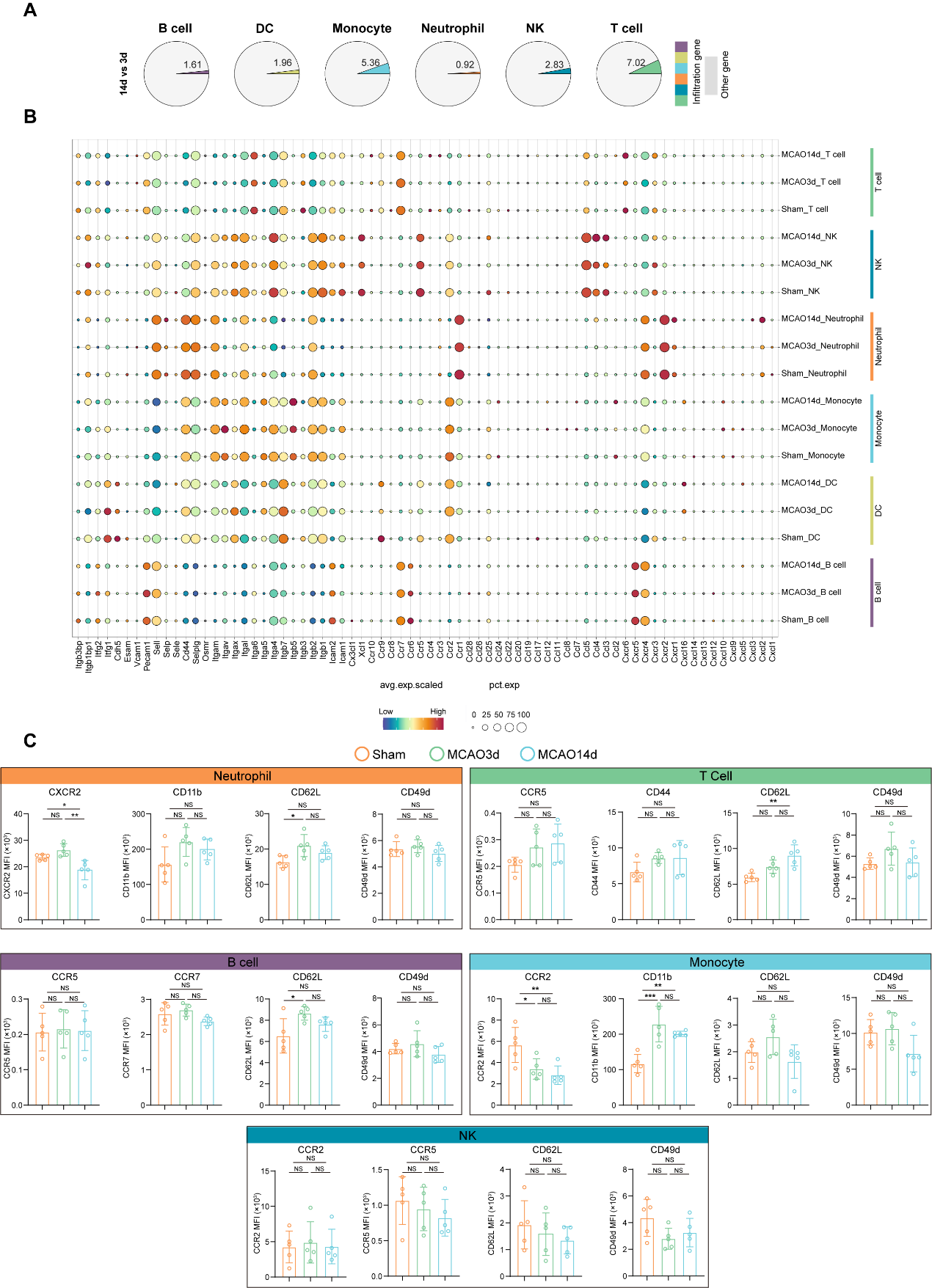


**Figure S2.**

**A)** Proportion of infiltration-related genes among upregulated genes in peripheral immune cells at MCAO day 14 versus day 3.

**B)** Dot plot displaying the expression differences of immune-related genes across different groups in the six cell clusters.

**C)** Flow cytometric analysis of infiltration-related molecule expression on peripheral immune cells across different groups. *P-value < 0.05, **P-value < 0.01, ***P-value < 0.001, NS means not significant. ONE-Way ANOVA. n = 5 per group.


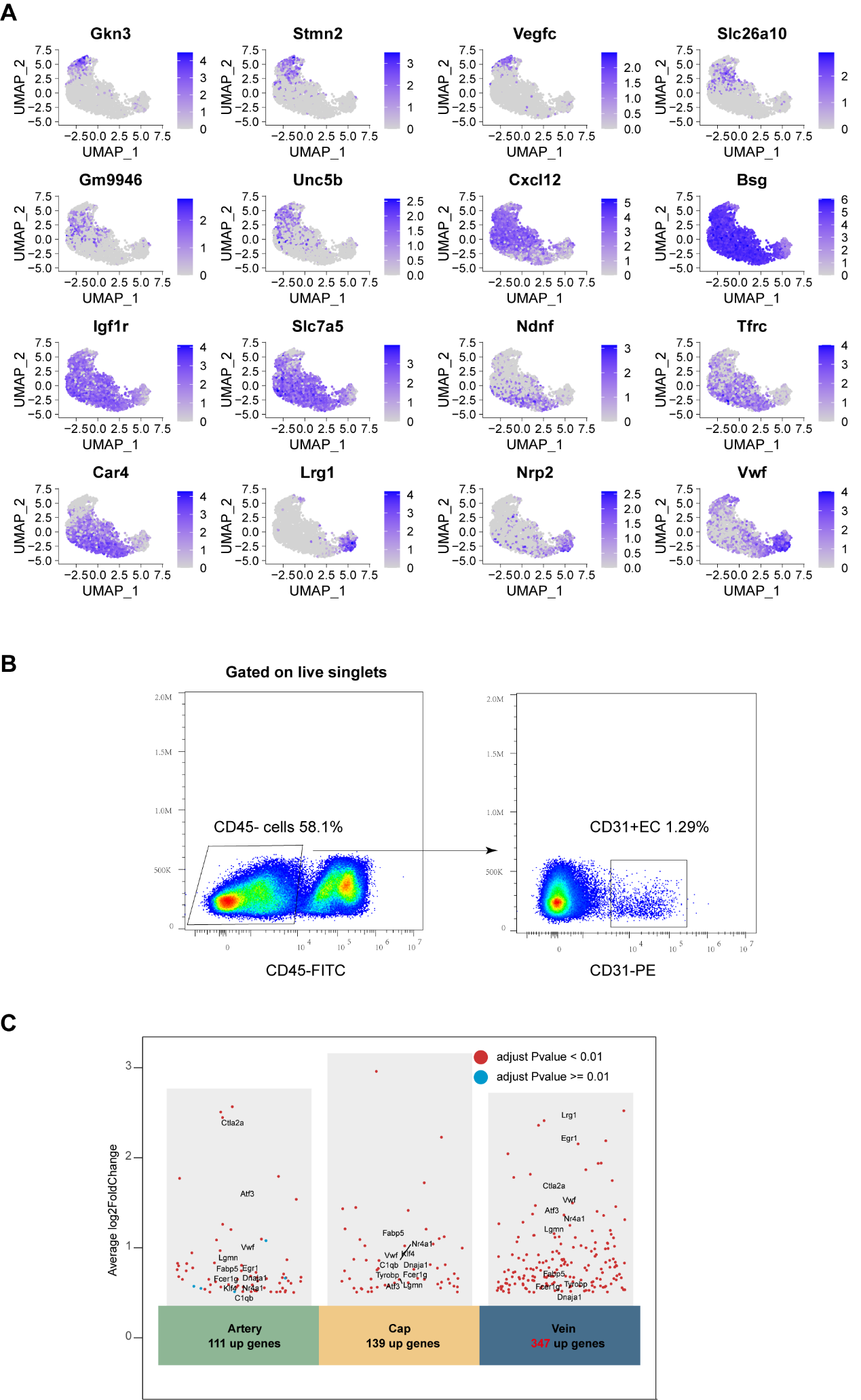


**Figure S3.**

**A)** Feature plot showing the expression of marker genes in the five ECs clusters.

**B)** Flow cytometry gating strategy for ECs of mouse brain.

**C)** Scatter plot showing upregulated gene numbers in three types of ECs (arteries, veins, and capillaries). The y-axis displays the log2(fold change).


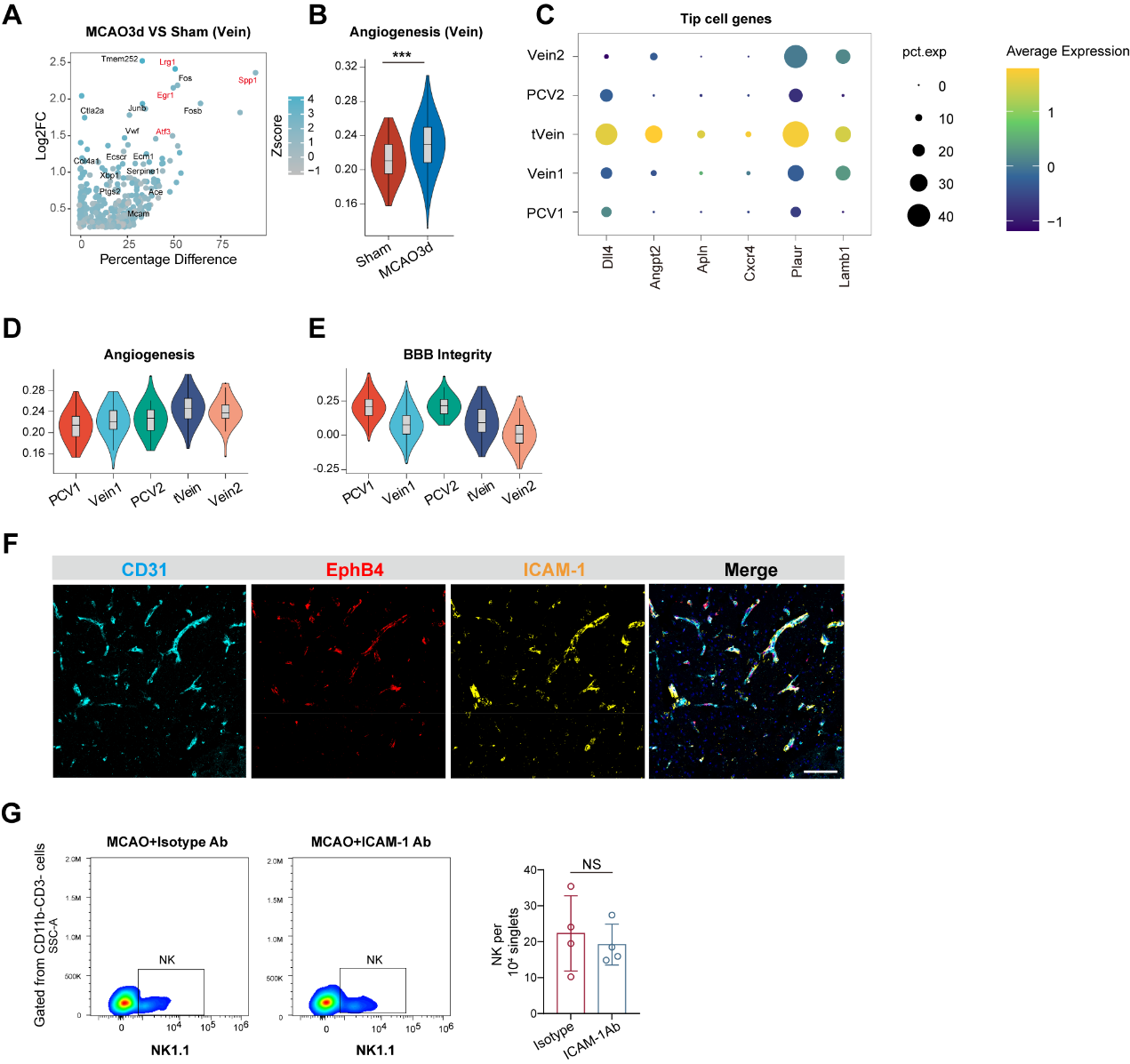


**Figure S4.**

**A)** Scatter plot showing the upregulated genes with a log2(fold change) > 0.25 in the venous ECs of the MCAO 3d group compared to the Sham group, with the percentage difference along the x-axis and log2(fold change) along the y-axis. The combined z-score of percentage difference and log2(fold change) is shown in the color scale.

**B)** Violin plot showing the difference in angiogenesis function between venous ECs of MCAO 3d and Sham groups. *** P-value < 0.001. Bonferroni-corrected Wilcoxon rank sum test.

**C)** Dot plot displaying the expression of tip cell genes in venous EC subpopulations.

**D-E)** Violin plots showing the differences in angiogenesis **(D)** and BBB integrity **(E)** among the venous EC subpopulations.

**F)** Immunofluorescence staining showing the expression of CD31 (blue), EphB4 (red), and ICAM-1 (orange) in the brain at 3 days post-MCAO.

**G)** Flow cytometry results showing no significant difference in NK cell infiltration between the Isotype and ICAM-1 block groups. NS means not significant. Mann-Whitney test. n = 4 per group.


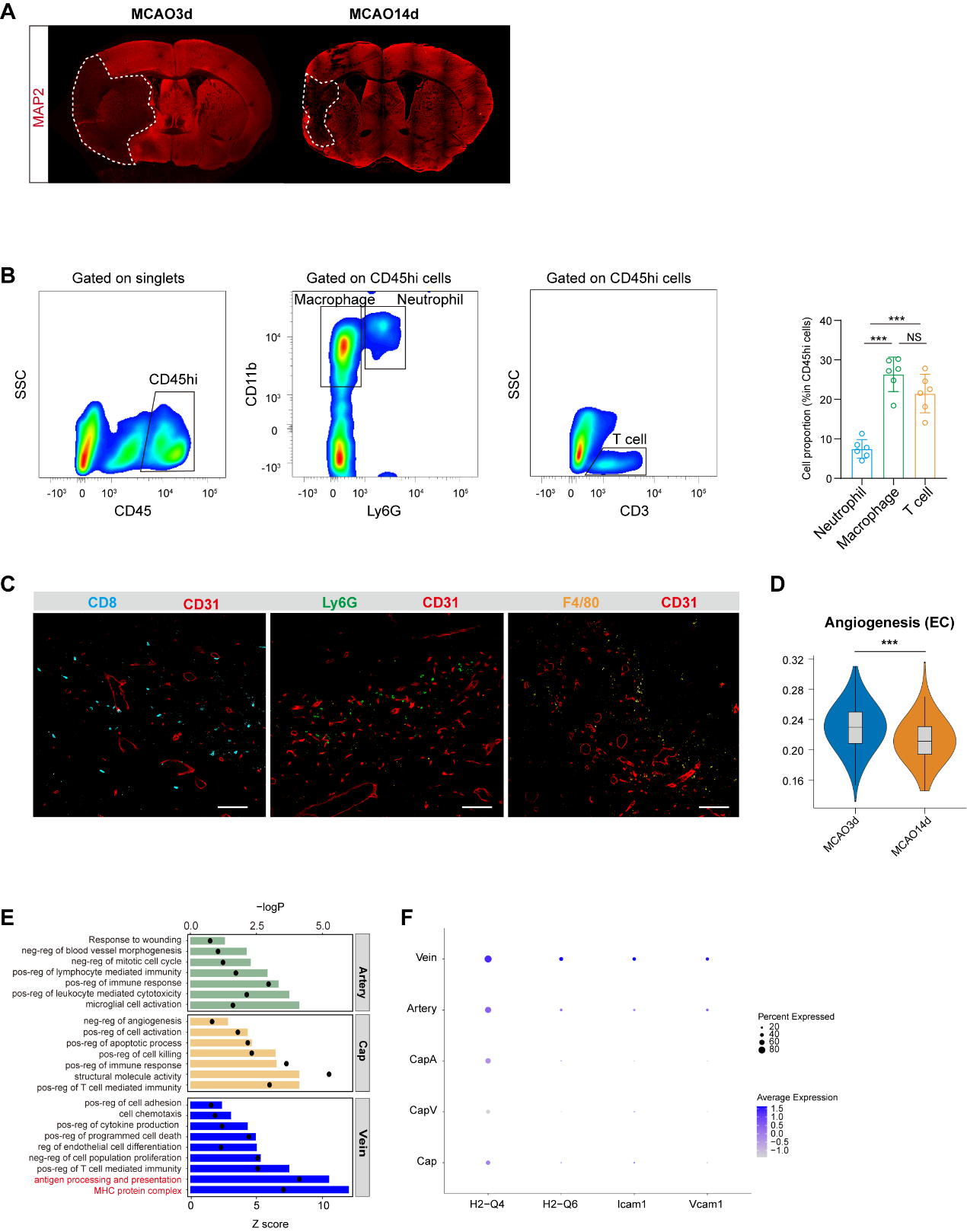


**Figure S5.**

**A)** MAP2 staining showing the infarcted areas outlined by white dashed lines in MCAO mice at day 3 and day 14.

**B)** Flow cytometry gating and proportion of brain neutrophils, macrophages, and T cells. ***P-value < 0.001, NS means not significant. ONE-Way ANOVA. n = 6 per group.

**C)** Representative images of immunofluorescence staining of CD8+T cells (blue) or neutrophils (green) with ECs (red) at 14 days post-MCAO. Scale bar: 100 µm.

**D)** Violin plots showing the differences in angiogenesis between the MCAO 3d and MCAO 14d groups. *** P-value < 0.001. Bonferroni-corrected Wilcoxon rank sum test.

**E)** Enrichment analysis of characteristic genes in three types of ECs (arteries, veins, and capillaries) at MCAO 14d. Bars represent z-score values, and dots represent significance (-log10[adjusted P-value]).

**F)** Dot plot showing high expression of antigen presentation and adhesion molecule genes in venous ECs at 14 days post-MCAO.


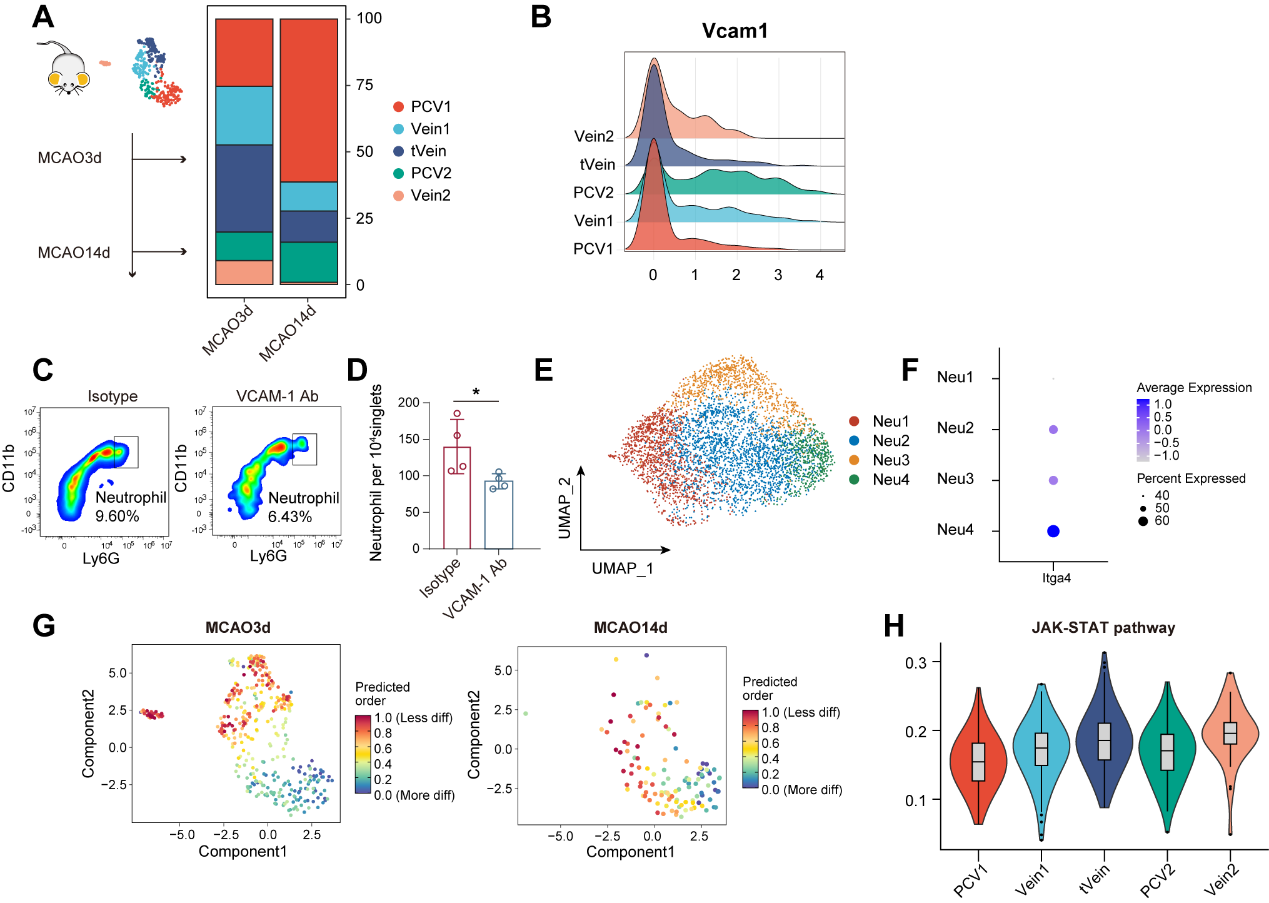


**Figure S6.**

**A)** Stacked bar plot showing the proportion of venous ECs clusters in the MCAO 3d and MCAO 14d groups.

**B)** Ridge plot displaying the expression of *Vcam1* in each venous ECs subpopulation.

**C-D) (C)** Flow cytometry comparison of the proportion of infiltrated neutrophils between the Isotype and VCAM-1 blockade groups; **(D)** VCAM-1 blockade leads to a reduction in the infiltration of neutrophils. *P-value < 0.05. Mann-Whitney test. n = 4 per group.

**E-F) (E)** UMAP plot of peripheral neutrophil subsets at 14 days post-MCAO; **(F)** Dot plot showing that at 14 days post-MCAO, Neu4 subset highly express *Itga4*.

**G)** Differentiation degree of venous ECs at 3 days and 14 days post-MCAO. Red represents lower differentiation, while blue represents higher differentiation.

**H)** Violin plot showing the expression of JAK-STAT pathway-related genes among the venous ECs.
